# Current Concepts in the Treatment of Distal Femur Fractures in Adults

**DOI:** 10.1055/s-0046-1822639

**Published:** 2026-06-08

**Authors:** Fabricio Fogagnolo, Ricardo Antônio Tavares

**Affiliations:** 1 Department of Orthopedics and Anesthesiology, Hospital das Clínicas, Faculdade de Medicina de Ribeirão Preto, Universidade de São Paulo, Ribeirão Preto, SP, Brazil

**Keywords:** femoral fractures, Hoffa fracture, knee, osteoporosis, fraturas de Hoffa, fraturas do fêmur, joelho, osteoporose

## Abstract

Distal femur fractures account for approximately 1% of all fractures and 3 to 7% of femoral fractures, representing complex injuries that frequently affect articular surfaces. Computed tomography is essential to evaluate articular involvement, the presence of coronal fracture lines, and metaphyseal comminution. Surgical treatment aims to restore mechanical alignment and articular congruity while providing sufficient stability to allow early mobilization.

The epidemiology shows a bimodal pattern: in younger patients, high-energy trauma predominates, whereas in older patients, low-energy falls are more common, with higher mortality related to osteoporosis and comorbidities. Conservative treatment has limited indications and is often reserved for patients with high surgical risk or for nondisplaced fractures.

Surgical management includes locking plates, retrograde intramedullary nails, and combined fixation techniques. Dual fixation has gained increasing attention in osteoporotic bone, cases with loss of medial support, and periprosthetic fractures. In selected cases, particularly in older patients with severely compromised bone quality, knee arthroplasty may be an alternative for early mobilization. The present article presents an updated review of the main epidemiological, diagnostic, and therapeutic aspects of distal femur fractures, including special situations such as open fractures, Hoffa fractures, and periprosthetic fractures.

Complications such as infection, nonunion, stiffness, and posttraumatic osteoarthritis remain frequent.

## Resumo

As fraturas distais do fêmur representam aproximadamente 1% de todas as fraturas e 3 a 7% das fraturas femorais, caracterizando-se como lesões complexas que frequentemente envolvem a superfície articular. A tomografia computadorizada é fundamental na avaliação da extensão articular, de eventuais traços coronais e da cominuição metafisária. O tratamento cirúrgico busca restaurar o alinhamento mecânico, a congruência articular e promover estabilidade suficiente para mobilização precoce.

A epidemiologia apresenta padrão bimodal: em jovens, predominam traumas de alta energia; em idosos, prevalecem quedas de baixa energia, com maior mortalidade devido à osteoporose e às comorbidades. O tratamento conservador possui indicações restritas, sendo normalmente reservado a pacientes com alto risco cirúrgico ou fraturas não desviadas.

O tratamento cirúrgico inclui placas bloqueadas, hastes intramedulares retrógradas e técnicas combinadas. A dupla fixação tem ganhado destaque em ossos osteoporóticos, perda de suporte medial e fraturas periprotéticas. Em casos selecionados, especialmente em idosos com osso muito fragilizado, a artroplastia do joelho pode ser uma alternativa para possibilitar mobilização precoce. Este artigo apresenta uma revisão atualizada dos principais aspectos epidemiológicos, diagnósticos e terapêuticos das fraturas distais do fêmur, incluindo situações especiais, como as fraturas expostas, fraturas de Hoffa e as fraturas periprotéticas.

Complicações como infecção, pseudoartrose, rigidez e osteoartrite pós-traumática ainda são frequentes.

## Introduction

Distal femur fractures account for approximately 1% of all fractures and 3 to 7% of femoral fractures. 1 2 Computed tomography (CT) plays a fundamental role in their evaluation, as it identifies posterior coronal fracture lines, determines the degree of metaphyseal comminution, and helps guide the choice of surgical strategy. Surgical planning, in turn, should consider restoration of the mechanical alignment in both the coronal and sagittal planes, anatomical reconstruction of the articular surface, and the achievement of sufficient stability to allow early mobilization. The introduction of locking plates, retrograde intramedullary nails, and combined fixation techniques 3 4 5 6 has significantly expanded the therapeutic arsenal, enabling improved fixation in complex fracture patterns even in cases of poor bone quality, while allowing weight bearing appropriate to the patient's clinical status. 7 8 9 Despite these advances, relevant complication rates persist, including stiffness, nonunion, implant failure, infection, and posttraumatic osteoarthritis.

## Epidemiology and trauma mechanisms

Distal femur fractures present a clearly bimodal epidemiological distribution. 2 In young adults, high-energy trauma predominates. These patients often present open fractures, complex ligament injuries, traumatic knee dislocations, neurovascular compromise, and associated injuries, significantly increasing the risk of complications. 10 The magnitude of the energy involved also contributes to a higher incidence of complete articular fractures, including posterior coronal fracture lines (Hoffa fractures). 11 In older patients, the predominant mechanism is low-energy trauma, usually resulting from ground-level falls (accounting for 61% of cases). 1 In this group, fractures are more prevalent among women. The presence of osteoporosis, sarcopenia, and multiple comorbidities contributes to fracture patterns characterized by metaphyseal comminution, cortical bone fragility, and an increased risk of fixation failure. Mortality in this population is high and may reach 26 to 30% within the 1 ^st^ year after the fracture, 3 12 13 14 15 a rate comparable to that observed in hip fractures. In low-energy trauma among older patients, the central challenge is the early surgical treatment of those with multiple comorbidities while allowing joint mobilization without increasing the risk of implant failure. This scenario reinforces the importance of an individualized approach to treatment selection.

## Diagnosis

The diagnosis of distal femur fractures requires a systematic approach. Inspection may reveal deformity, limb shortening, external rotation, edema, and, in high-energy trauma cases, open fractures or indirect signs of associated joint injuries. Neurovascular evaluation is mandatory given the proximity of the popliteal structures. Arterial injuries may present as absent pulses, reduced capillary refill, a cold extremity, or even subtle asymmetry in the posterior tibial and dorsalis pedis pulses. In suspected cases, Doppler evaluation, CT angiography, or immediate consultation with vascular surgery is essential. When open, these fractures are associated with a worse prognosis due to the increased risk of infection, a higher number of secondary surgical procedures, and higher rates of delayed union and nonunion. Partial or complete injuries of the quadriceps tendon may also occur, usually caused by direct injury from the proximally displaced fragment.

Initial anteroposterior (AP) and lateral radiographs are indispensable and allow identification of the general fracture pattern; however, complete fracture assessment requires CT to evaluate intraarticular extension (approximately 55% of these fractures are intraarticular 16 ), detect possible occult fracture lines, and ensure accurate identification of Hoffa fractures.

A complete diagnosis should integrate all these elements, ensuring that the surgeon understands the osseous fracture pattern, the condition of the soft tissues, and the presence of associated injuries that may influence the therapeutic strategy.

## Classification

The Arbeitsgemeinschaft für Osteosynthesefragen/Orthopedic Trauma Association (AO/OTA) system is widely used for classifying distal femur fractures. In this system, type A fractures are extraarticular, type B fractures are partial articular, and type C fractures are complete articular injuries, in which there is no continuity between the diaphysis and the articular surface. Fractures of the distal femur can have a significant impact on the knee joint, as even relatively small deviations in the coronal or sagittal planes may substantially affect the mechanical axis of the limb and potentially result in post-traumatic osteoarthritis in the long term.

## Indications for conservative treatment

Currently, conservative treatment is reserved for a very limited group of patients, and the literature provides few comparisons between surgical and nonoperative management. 17 With the evolution of osteosynthesis techniques, nonoperative treatment has become the exception, and recent studies have shown lower mortality with surgical treatment in elderly patients. 18

Except in the pediatric population, in which conservative treatment with casting or traction may be indicated in selected situations, there are few circumstances in which surgical treatment is not indicated in adult patients. These include elderly patients who are non-ambulatory, those with high surgical risk due to comorbidities, patients with active infection in the affected limb, and cases of nondisplaced fractures. Even in those cases, external fixation might be considered. Immobilization of femoral fractures with splints, braces, or external supports is extremely difficult. Therefore, surgical treatment also plays a significant role in promoting patient comfort and reducing both systemic and local complications, even in patients with limited functional status.

## Surgical treatment

### Timing of surgery

Determining the optimal timing for surgery requires careful evaluation of the patient's clinical condition, the status of the soft tissues, and the presence of associated injuries. In patients with isolated fractures, definitive treatment can usually be planned and performed within the first few days after injury. In high-energy trauma, patients frequently present with soft-tissue injuries or significant edema, and temporary external fixation is a common stabilization strategy. In polytraumatized patients, definitive osteosynthesis occurs once the patient reaches the so-called “window of opportunity,” typically 5 to 10 days after the trauma, when hemodynamic and metabolic parameters have stabilized. In elderly patients with low-energy fractures, prolonged surgical delay may result in higher complication rates, and delays exceeding 2 or 3 days significantly increase morbidity and mortality. 3 14 As these are complex fractures, careful preoperative evaluation of imaging studies and the patient's clinical status is required for adequate surgical planning. Surgical planning involves determining the optimal timing of surgery and selecting the surgical approaches, reduction strategies, implants, and fixation methods.

## Surgical planning

Patient positioning varies according to the surgical approach and the fracture pattern. Although the supine position is the most common, posterior Hoffa fractures may require alternative positioning or combined approaches. In some cases, patient positioning may require modification during surgery, with conversion to the floating position. The surgical exposure must allow direct visualization and reduction of the articular fracture lines, as well as indirect reduction of the metaphysis. Distractors and periarticular clamps facilitate fracture reduction and should be readily available for use. The articular surface should be anatomically reduced and stabilized with interfragmentary compression, whereas the metaphysis requires relative stability. Distal screws should not violate the Blumensaat line (intercondylar region), and radiographic views with 25° of internal rotation allow proper assessment of screw length in the medial condyle. The potential need for bone grafting should be considered during surgical planning. Historically, its use has been recommended in cases of significant bone defects or absence of medial cortical contact. More recently, with the advent of fixation using a second medial implant, there is no formal recommendation for routine bone grafting. A second medial implant has been indicated in osteoporotic bone, periprosthetic fractures, or when medial bone contact is absent. 7 Coronal and sagittal alignment should be repeatedly checked with fluoroscopy, 19 using the normal metaphyseal angles and the contralateral limb as references. Since these procedures are often lengthy, avoid tourniquet use. However, a tourniquet may be positioned proximally and inflated only if necessary, without interfering with the placement of proximal screws.

## Approaches and reduction and fixation techniques

### Extra-articular fractures (AO 33A)

The treatment of extraarticular fractures (AO 33A) can involve either plates or intramedullary nails—the latter is particularly advantageous due to indirect reduction and percutaneous insertion. Since there is no joint involvement, the primary objective is to restore coronal, sagittal, and axial alignment, as well as limb length. The action of the gastrocnemius muscles may require counteraction using a bolster placed under the knee or a Steinmann pin inserted from anterior to posterior into the distal fragment and pulled distally. This maneuver tensions the posterior capsule and prevents recurvatum deformity. This technique, described by Paccola, 20 should be performed without the knee bolster to avoid antecurvatum. Plate fixation requires strict adherence to anatomical parameters to avoid violation of the intercondylar region, the patellofemoral joint, or undue medialization of the condylar block. Implants such as dynamic condylar screws (DCS, Synthes) or locking plates should align with the femoral shaft on the lateral view. Distal screws must remain anterior to the Blumensaat line. To minimize friction with the iliotibial band, the implant should be positioned with 10° internal rotation, adapting to the lateral wall of the condyle. Angled blade plates, such as the 95° blade plate, are currently used mainly for osteotomies and have largely been replaced by anatomical locking plates in the setting of acute trauma.

In retrograde intramedullary fixation, a small longitudinal anterior incision provides access to the entry point, which should align with the medullary canal and be approximately 1 cm anterior to the intercondylar notch roof. Maintenance of reduction may require multiple distal locking screws and strategic placement of poller screws. The distal end of the nail should not protrude to avoid patellofemoral impingement. 9

### Unicondylar articular fractures (AO 33 B1 and B2)

Unicondylar articular fractures require a surgical approach that allows direct visualization of the articular fracture line. This pattern frequently presents with sagittal shear and a wedge fragment with a well-defined metaphyseal apex. In such cases, in addition to lag screws placed perpendicular to the fracture line, absolute stability is achieved with the placement of a buttress plate at the apex, preventing secondary loss of reduction under axial load. Wedge fractures are well suited for indirect reduction, in which a pre-contoured plate positioned along the shear plane achieves fracture reduction during the insertion of the first screw through the hole adjacent to the apex.

**Lateral fractures:** Simple lateral unicondylar fractures require a direct lateral approach, with an anterolateral extension toward the tibial tuberosity. Depending on the location of the fracture line, the anterolateral approach may provide superior visualization, particularly in the presence of articular impaction. Plate insertion may be submuscular, while proximal screws placement is percutaneous.

**Medial fractures:** For medial unicondylar fractures, the medial subvastus approach provides excellent exposure, including for coronal (Hoffa) fractures. Typically, reduction is achieved under traction, with the knee in extension and application of valgus stress (or varus stress for lateral fractures).

Impaction or more oblique fracture planes may require posteromedial or posterolateral approaches. 11

### Complete articular bicondylar fractures (AO 33C)

The most common complete articular fractures are AO types C2 and C3, which present with metaphyseal comminution. The DCS introduced the advantage of metaphyseal angular stability and remains useful in selected cases. There is also a high incidence of coronal Hoffa-type fracture lines (approximately 38%). 9 11 21 In these cases, anatomical locking plates are preferred because their multiple distal screw holes provide greater flexibility for condylar fixation while avoiding conflicts with screws placed in other directions during fixation of the Hoffa fragment.

**Fig. 1:**
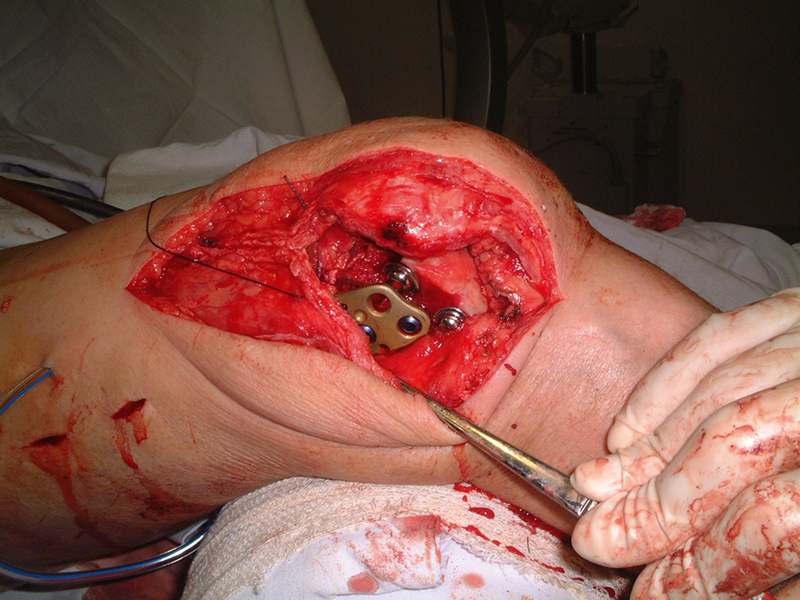
Intraoperative image showing 6.5-mm lag screws strategically placed at the periphery of the lateral femoral condyle, avoiding the region of subsequent positioning of the lateral plate. Anterior transarticular approach and retrograde plate osteosynthesis (TARPO), lateral parapatellar approach. The current trend is to use 3.5-mm lag screws, which occupy less space, using the compression technique with a gliding hole.

The anterolateral approach (transarticular approach and retrograde plate osteosynthesis [TARPO]) 22 provides wide articular exposure and allows plate fixation through the same approach in most cases. The anteromedial approach is preferred when medial comminution predominates and combined medial-lateral fixation may be used when necessary. With anterior approaches, osteotomy of the tibial tuberosity—previously performed in complex cases through the lateral approach—has become unnecessary. Articular surface reduction uses Kirschner wires, with small-fragment screws placed peripherally to allow proper positioning of the lateral plate. Cortical screws with 3.5mm in diameter are preferred because they occupy less space than cancellous 6.5-mm screws. It is important to ensure that long 3.5-mm screws are available, as some instrument sets provide screws with a maximum length of 50 mm. Whenever possible, avoid penetrating the cartilage. When this is unavoidable, the entry point should be countersunk to bury the screw head; alternatively, small headless screws can be used. Smaller osteochondral fragments may require minifragment implants and horizontal plates. 23 24 After articular fixation, indirect metaphyseal reduction is performed under fluoroscopy while preserving vascularization as much as possible ( Fig. 1 ). Coronal and sagittal alignment should be repeatedly checked using the mechanical axis under fluoroscopy. 19 The most common deformity is valgus associated with recurvatum.

Currently, retrograde intramedullary nails with multiple distal locking options and lateral locking plates are the most used implants. 4 7 9 25 26 27 Retrograde nails are appropriate for 33C1–C2 fractures, but they are riskier in 33C3 fractures due to articular fragmentation. Alignment control with nails is more difficult and may require poller screws. Small plates for metaphyseal reduction may also complement fixation when intramedullary nails are used. In elderly patients, long implants are preferred to protect the entire bone length.

Recent meta-analyses report similar outcomes between nails and plates regarding union rates, complications, reoperations, and operative time, with small differences favoring plates in terms of mobility and quality of reduction, although with higher rates of bone healing disturbances. 8 28 The choice of implant should consider the complexity of the articular fracture pattern, the difficulty of reduction control (where plates may be superior), the need for early weight bearing (favoring nails), and the patient's clinical status.

### Double implant

**Fig. 2:**
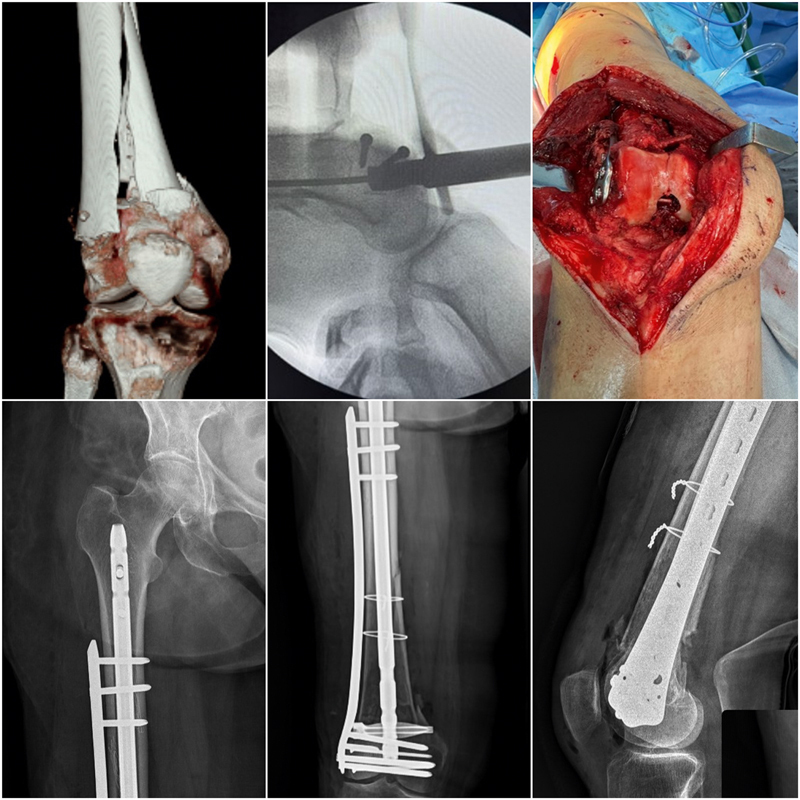
Intraoperative images of a distal femur fracture in osteoporotic bone in a 69-year-old patient, transferred 9 days after the initial trauma, with provisional fracture fixation. Note the reduction strategy using the anterior transarticular approach and retrograde plate osteosynthesis (TARPO) method, with fixation of the articular fracture employing screws positioned outside the trajectory of the long intramedullary nail, which extends beyond the level of the lesser trochanter. Lastly, the lateral plate is adjusted with screws to supplement the articular fixation, with screws diverging from the proximal region of the nail. The use of two steel cerclage wires in the diaphysis facilitated fracture reduction and increased fixation stability.

Double-implant osteosynthesis—consisting of a lateral plate combined with a medial plate, or a medial or lateral plate combined with a retrograde intramedullary nail—has gained increasing attention in situations with no medial contact or support, osteoporotic bone, obese patients, poorly compliant patients (alcoholism, dementia, or drug abuse), and periprosthetic fractures. 4 5 6 26 29 30 31 Dual plate fixation offers better control of reduction quality but at the cost of greater surgical dissection, slightly increased blood loss, and higher rates of bone healing problems. The nail–plate combination provides greater mechanical stability, facilitating early weight bearing and potentially resulting in higher union rates, including in cases of nonunion. 5 6 The combined use of nails and plates incorporates the advantages of both methods. Plates provide superior torsional resistance and improved control of articular fracture reduction, whereas nails offer biomechanical advantages due to their central position and greater axial stability. This combination makes early full weight bearing safer, which is particularly beneficial in elderly patients. However, the use of both implants requires surgical expertise and careful screw placement to avoid conflict with the nail, which is usually inserted before plate fixation. The typical sequence involves reduction and fixation of the articular fragments followed by nail insertion and then the plate afixation ( Fig. 2 ).

### Knee arthroplasty as an alternative to fixation

In elderly patients with osteoporotic bone and highly comminuted fractures, or in certain periprosthetic fractures with loosening of the femoral component, complication rates after osteosynthesis—such as fixation failure, nonunion, and reoperation—are considerable. In these situations, when successful fixation is considered unlikely, knee arthroplasty—either primary total knee arthroplasty or distal femoral replacement 32 33 —may be considered as an alternative to allow early mobilization, weight bearing, and reduced immobilization time, which are crucial factors in patients with multiple comorbidities. The prosthetic implants used in these cases often have greater constraints to compensate for the ligamentous instability resulting from the fracture. When loosening of the femoral component is present, complete replacement of the distal femur may be an option. However, these specialized implants may have limited availability, and the procedures are technically more demanding and costlier. Ideally, the treatment of these cases should occur in centers with experience in complex arthroplasty and access to modular prostheses and metaphyseal augments. Complication rates and mortality are naturally higher than in patients undergoing primary knee arthroplasty for osteoarthritis. Recent reviews also show that arthroplasty is associated with more clinical complications than internal fixation. 34 35

## Special situations

### Open fractures

Open fractures account for 11 to 14% of low-energy distal femur fractures and up to 55% of high-energy fractures. These injuries are associated with a higher risk of infection, fixation failure, and healing issues. 36 They require immediate management, following the established principles for open fractures, including early antibiotic prophylaxis, operative debridement, and prompt soft-tissue coverage of the joint to prevent infection. Minimal articular fixation with screws may facilitate subsequent reconstruction, provided it does not compromise definitive fixation. Screw trajectories should not interfere with future plate placement or retrograde intramedullary nail insertion, and additional surgical approaches should be minimized to prevent further devascularization.

**Fig. 3:**
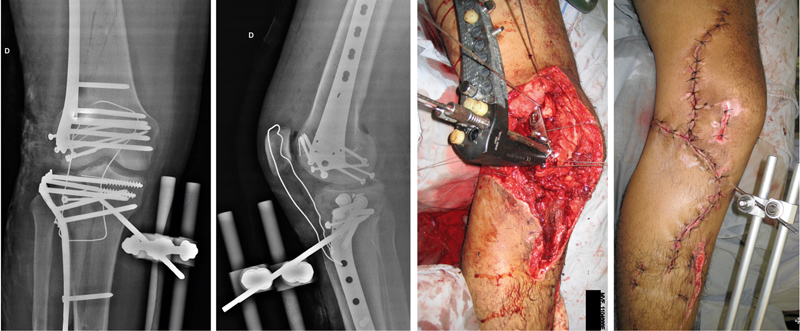
Osteosynthesis of an open 33C3 bicondylar fracture, showing the peripheral placement of 3.5-mm small-fragment screws (also fixing the lateral Hoffa component) and a locking plate. Primary skin closure remained feasible despite the high-energy trauma and soft-tissue injury.

Initial fixation should be rapid and performed with small-fragment screws through the same approach used for debridement, ideally by the surgeon responsible for the definitive procedure. Additional strategies include local antibiotic therapy with polymethyl methacrylate beads, vacuum-assisted dressings, and the use of cement spacers when the Masquelet technique is planned for metaphyseal bone defects. 37 The prognosis of open fractures is worse, with higher rates of infection, healing disturbances, and reoperations ( Fig. 3 ).

### Hoffa fractures

Busch-Hoffa fractures are common in complex bicondylar fractures, 38 although they may also occur in isolation. They occur in more than 30% of type-C fractures and may be missed in up to 25% of cases. 11 The coronal fracture line requires specific surgical approaches when fragments are more posterior or there are small osteochondral fractures. In addition to the typical shear wedge fragment, areas of chondral depression with interposed fragments may also be present. Hoffa fragment fixation typically uses anterior-to-posterior lag screws when associated with complex fractures. Reduction is facilitated by extension of the distal fragment during reduction of the condylar mass through the same anterior approach. In small posterior fragments, screws inserted from posterior to anterior provide greater stability. Fragments with metaphyseal extension should be supported with buttress plates to resist axial loads, using posterolateral or posteromedial approaches. Small-fragment plates positioned horizontally may also increase stability. 11 24

**Fig. 4:**
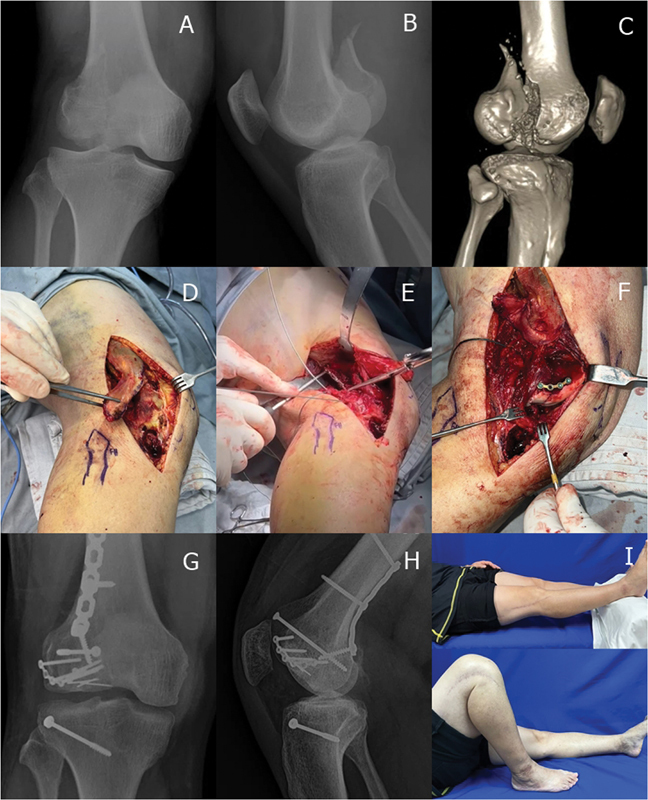
Coronal fracture of the lateral femoral condyle (Hoffa fracture) ( **A** , **B** ); Letenneur type-I variant ( **C** – intercalated fragment). In this case, a posterolateral approach was performed with the patient in the lateral decubitus position. Osteotomy of the Gerdy tubercle ( **D** ) allowed direct visualization of the articular fragmentation as well as the metaphyseal region for placement of the buttress plate ( **E** ). Note the use of a small minifragment plate positioned horizontally ( **F** ) to improve stability of the articular surface. Postoperative radiographs ( **G** , **H** ); joint mobility 5 months after surgery ( **I** ).

The Letenneur classification 11 assists in surgical planning:

Type I: fracture line aligned with the posterior cortex; large fragments. A variant includes comminution or articular depression. The most stable fixation involves a 3.5-mm buttress plate at the fracture apex in addition to screws inserted from posterior to anterior, preferably through a posterolateral approach, sparing the fibular nerve. In variants with depressed fragments, a lateral approach combined with osteotomy of the Gerdy tubercle allows visualization and reduction of these fragments ( Fig. 4 ).

Type II: smaller and more posterior fragments, subdivided into IIa, IIb, and IIc according to size. Because these fragments are more posterior and smaller, posterior approaches are usually required, and screw insertion often occurs through the cartilage.

Type III: a more oblique and anterior articular fracture line. These fractures are usually approached anterolaterally, with fixation from anterior to posterior.

Isolated fractures of the medial condyle are less common. The medial subvastus approach is usually preferred; rare cases may require a posterior approach between the medial gastrocnemius and the gracilis muscles.

### Periprosthetic fractures

Periprosthetic fractures occur in 0.3 to 5.5% of primary arthroplasties and in up to 30% of revision procedures. 29 39 Most occur after low-energy trauma and are more common in elderly patients and in women. Other risk factors include corticosteroid use, comorbidities, malalignment, and intraoperative technical errors. More recently, fractures associated with robotic navigation pin sites have also been reported.

In periprosthetic knee fractures, osteoporosis and limited bone stock make fixation more complex, particularly when the femoral component has a closed intercondylar box, a feature present in some posterior-stabilized prostheses. Assessment of component stability is key, and CT with metal artifact reduction is a critical diagnostic tool. Prosthetic revision is mandatory in cases with loosening; in the United Kingdom, approximately 3.8% of revision procedures result from these fractures.

The Unified Classification System (UCS) has gained increasing adoption, although the Lewis-Rorabeck classification remains the most used: 30

- Type I: nondisplaced fracture, stable prosthesis;
- Type II: displaced fracture, stable prosthesis; and
- Type III: loosening of the femoral component.

**Fig. 5:**
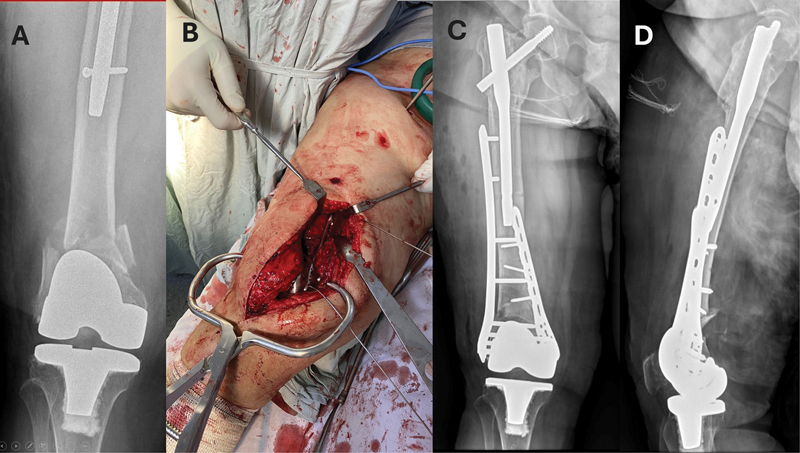
Interprosthetic fracture of the distal femur ( **A** ) fixed with two locking plates. The intraoperative photograph ( **B** ) illustrates the medial approach and reduction using a large periarticular clamp. A more extensive anteromedial approach was performed, along with a second lateral approach for percutaneous insertion of the lateral plate. The following panels show the radiographs in anteroposterior (AP) ( **C** ) and lateral ( **D** ) views. It is important to avoid positioning the plates so that they terminate at the same level or create a stress concentration zone between the nail and the plates. Special periprosthetic screws were used at the most proximal part of the lateral plate.

In typeI and -II fractures, there is a trend toward the use of dual plating or plates combined with intramedullary nails, employing long implants that protect the entire femoral length. The lateral plate is inserted through a direct lateral approach using a submuscular technique, with proximal screws placed percutaneously. The medial plate is usually smaller and inserted through a medial subvastus approach ( Fig. 5 ). When using a long medial plate, the femoral artery must be protected by means of twisting the proximal part of the plate. In selected cases, a long retrograde intramedullary nail may be used alone or combined with lateral plates. It is essential to confirm beforehand whether the femoral prosthetic component allows passage of a retrograde nail (open-box design). A relatively common issue is posterior displacement of the nail entry point due to the design of the femoral component, potentially leading to recurvatum deformity. To address this limitation, specific retrograde intramedullary nails for periprosthetic fractures have been developed with greater distal angulation, allowing a more posterior entry point without inducing recurvatum.

In type III fractures, when prosthetic loosening is present, long-stem prosthetic femoral components may be used, preferably combined with a lateral plate. In cases of severe comminution or extensive bone loss, distal femoral replacement prostheses or revision with structural allografts may be considered if available.

## Rehabilitation

Early joint mobilization should be encouraged, and modalities such as continuous passive motion (CPM) may assist in the progressive recovery of range of motion. Effective analgesia is essential for functional recovery. Antithrombotic prophylaxis is maintained for 10 to 30 days. Early ambulation using two crutches or a walker should be encouraged. Prolonged non-weight bearing is detrimental and may lead to dystrophy, vascular complications, and delayed fracture healing. Weight bearing as tolerated can be safely permitted in periprosthetic fractures treated with plates or intramedullary nails, 40 even in elderly patients. Dual-implant constructs further increase stability under axial load.

## Outcomes and complications

The functional outcomes of distal femur fractures vary depending on fracture pattern, presence of associated injuries, surgical technique, and quality of rehabilitation. When anatomical reduction and adequate stability are achieved, most patients recover sufficient joint mobility to perform activities of daily living. However, complex articular fractures frequently progress with some degree of stiffness and residual pain. The most feared complications are postoperative infection, healing disturbances, and fixation failure. In the long term, the risk of posttraumatic osteoarthritis should always be considered.

## Final considerations

Distal femur fractures remain challenging injuries, and the present paper aimed to summarize the main aspects involved in their treatment. Their bimodal epidemiological distribution includes young patients who are victims of high-energy trauma and elderly individuals who generally present with multiple comorbidities and a slower and more complex functional recovery. Despite advances in the development of modern intramedullary implants and anatomical locking plates, the morbidity and mortality associated with these fractures remain significant.

The current article discussed complex situations, including open, Hoffa, and periprosthetic fractures, all of which require different treatment strategies. Among current trends, the use of dual fixation deserves particular attention, as it has shown advantages in highly comminuted fracture patterns, in the absence of medial bone contact, in osteoporotic bone, and in cases of periprosthetic fractures.

Lastly, treatment success depends not only on bone stabilization but also on appropriate rehabilitation, including early weight bearing and joint mobilization, particularly in the elderly population.
